# Adaptation of Ritchie's Method for Parasites Diagnosing with Minimization of Chemical Products

**DOI:** 10.1155/2012/409757

**Published:** 2012-07-16

**Authors:** Régis Silva Anécimo, Karina A. A. Tonani, Brisa Maria Fregonesi, Ana Paula Mariano, Marinês D. B. Ferrassino, Tânia M. B. Trevilato, Roberta Braga Rodrigues, Susana I. Segura-Muñoz

**Affiliations:** ^1^Central Laboratory of Clinical Pathology, University Hospital, University of São Paulo at Ribeirão Preto Medical School, 14040-902 Ribeirão Preto, SP, Brazil; ^2^Laboratory of Ecotoxicology and Environmental Parasitology, University of São Paulo at Ribeirão Preto College of Nursing, Avenida Bandeirantes 3900, Campus Universitário, 14040-902 Ribeirão Preto, SP, Brazil; ^3^Microtechnics/Metal Sector, University of São Paulo at Ribeirão Preto Medical School Hospital das Clínicas, 14040-902 Ribeirão Preto, SP, Brazil

## Abstract

Latin America, Africa, and Asia present wide dissemination and high prevalence rates of waterborne parasitic diseases, which is a strong indicative of the fragility of public sanitation systems. In this context, parasitological analyses represent extremely relevant instruments. Several parasite diagnosis methods exist, among which Ritchie's method (1948) stands out. This method uses formaldehyde and ether, two reagents of toxicological importance that can cause damages to environmental and occupational health. The present study aimed to compare Ritchie's method modified by Régis Anécimo, without use of solvents, with the traditional Ritchie's method, routinely used for helminth and protozoa diagnosing in Brazil. Some changes were introduced in the modified method, such as controlled increase of water temperature used after stool dilution and substitution of formaldehyde and ether by a neutral detergent before material centrifugation for observation of parasites. In examined samples by both methods, multiple infections were commonly observed; the modified method presented a similar sensitivity to identify the parasites. The development of analytic diagnosis methods that minimize the use of chemical products like ether and formaldehyde represents an important tool to prevent occupational diseases among exposed professionals, as well as to preserve environmental quality through the use of clean techniques.

## 1. Introduction

In many regions, intestinal parasites represent a very important medical-sanitary problem, due to their large-scale dissemination and high prevalence. This situation is typical of developing countries, where interrelations between etiological agent and host are favored, considering the precarious basic sanitation conditions, predisposing environmental factors, and the unfavorable socioeconomic conditions of an important part of the population [1].

In developing countries, the most frequent helminthiases are those caused by intestinal infection with soil-transmitted helminths such as *Ascaris lumbricoides* and *Trichuris trichiura*, and hookworms (*Necator americanus and Ancylostoma duodenale*) [2]. According to Centers for Disease Control and Prevention (CDC) [3], around 1.5 billion individuals are infected with *Ascaris lumbricoides*, 1.1 billion with *Trichuris trichiura *and 1.3 billion with hookworms, and that, the main clinical manifestations are intestinal obstruction, malnutrition, and anemia due to iron deficiency. It is also estimated that 200 and 400 million people, respectively, host protozoa such as *Giardia duodenalis* and *Entamoeba histolytica,* causing diarrhea and poor absorption, whose clinical manifestations are usually proportional to the parasitical load hosted by the individual [4–6].

The clinical diagnosis of human parasites is generally accompanied by a laboratory diagnosis in order to confirm the suspicion. The parasitological analyses is an extremely relevant instrument in this field because it allows for the identification of parasites that live in the human digestive tube or use the stool as the normal vehicle for disseminating their forms to the external environment. In parasitology, the inclusion of different diagnosis methods depends on each parasite, considering the biological and morphological variability of the microorganism to be examined. There are several qualitative and quantitative methods for parasitological diagnosis, such as Spontaneous sedimentation method of Hofmman and Lutz; Centrifugal-flotation method of Faust; Staining methods of Ziehl Neelzen and Kato Katz and Sedimentation and centrifugation method of Ritchie [7, 8].

Among the methods mentioned above, Ritchie's method (1948) is highlighted, which is based on a methodology of acknowledged efficiency for the diagnosis of helminths and protozoa in stool through centrifugal-sedimentation in a formaldehyde ether system. Formaldehyde and the ether are two reagents of toxicological importance to both the environmental health as for occupational [9].

This study aimed to compare helminths and protozoa diagnose in stool using Ritchie's traditional method and a modified Ritchie's method, that avoid the use of toxic solvents.

## 2. Material and Methods

### 2.1. Place of Study

This study was performed at the Organic Fluids Section of the Central Laboratory of Clinical Pathology, University of São Paulo at Ribeirão Preto Medical School, Clinics Hospital, Ribeirão Preto, São Paulo, Brazil. It is considered the largest hospital in the interior of São Paulo State. Every year, the Laboratory of Clinical Pathology performs 2,676,500 laboratory exams (Diagnostic and Therapeutic Support), 354,700 specialized exams, and approximately 8,000 parasitological exams [10].

### 2.2. Sample Collection and Analysis Procedure

All patients who underwent the parasitological exam received a recipient to collect a fecal sample, labeled with their name and registration number. The patient collected the material at home and delivered it the following day at the collection room of the clinic hospital to be forwarded to the Organic Fluids Laboratory. A total of 392 samples were processed and examined by the Hoffmann Spontaneous Sedimentation Method [11] and Faust Zinc Sulfate Centrifugal Flotation Method [12] used for the identification of eggs, cysts, and larvae. For Hoffman method, approximately 2 g of fecal samples were homogenized in distilled water, filtered through gauze, and allowed to stand for 24 hours. Sediment was used for observation. For Faust method, approximately 2 g fecal samples were homogenized in distilled water (10 mL) and centrifuged at 2500 rpm during 1 min in conical tubes. Centrifugal process was repeated until solution become clear. Then, the solution was resuspended in ZnSO_4_ (33%) and centrifuged. Surface films were collected and observed. Both, sediment and surface film samples, were stained with lugol and observed in a microscope Nikon Eclipse E200. Positive samples (*n* = 150) were reserved for comparative analysis with Ritchie's method [9] and Ritchie's method Modified by Régis Anécimo, proposed in this study.

Ritchie's method modified by Régis Anécimo, is a soapy warm water centrifugal-sedimentation method. The technique is based on the suspension of approximately 2 g of stool in 10 mL of water at 45°C. The samples were homogenized and filtered through folded gauze with the help of a funnel (65 × 50 mm). Next, they were centrifuged at 2500 rpm during 1 min; supernatant material was removed. Sediment is resuspended with neutral detergent (100 *μ*L) in combination with 10 mL of water at 45°C, homogenized, and centrifuged at 2500 rpm during 1 min. A drop of the sediment was put on a blade with 1-2 drops of lugol and observed under an optical microscope Nikon Eclipse E200, with enlargement of 100x and 400x. To generate digital images, a microscope Zeizz AxioVision Scope A1 was used.

### 2.3. Statistical Analyses

Forstatistical analysis of the results, the Statistical Program Graph Pad Prism (Version 3.02 for Windows, Graph Pad Software, San Diego, CA, USA) was used. The Mann-Whitney nonparametric test was used. The level of significance was set at *P* = 0.05.

## 3. Results and Discussion

Table 1 shows the percentage of parasites found according to Ritchie's method and Ritchie's method Modified by Régis Anécimo. Multiple infections were commonly observed in the samples examined by both methods. The percentage of parasites observed in both techniques did not show statistical difference (*P* > 0.05).

**Figure 1:**
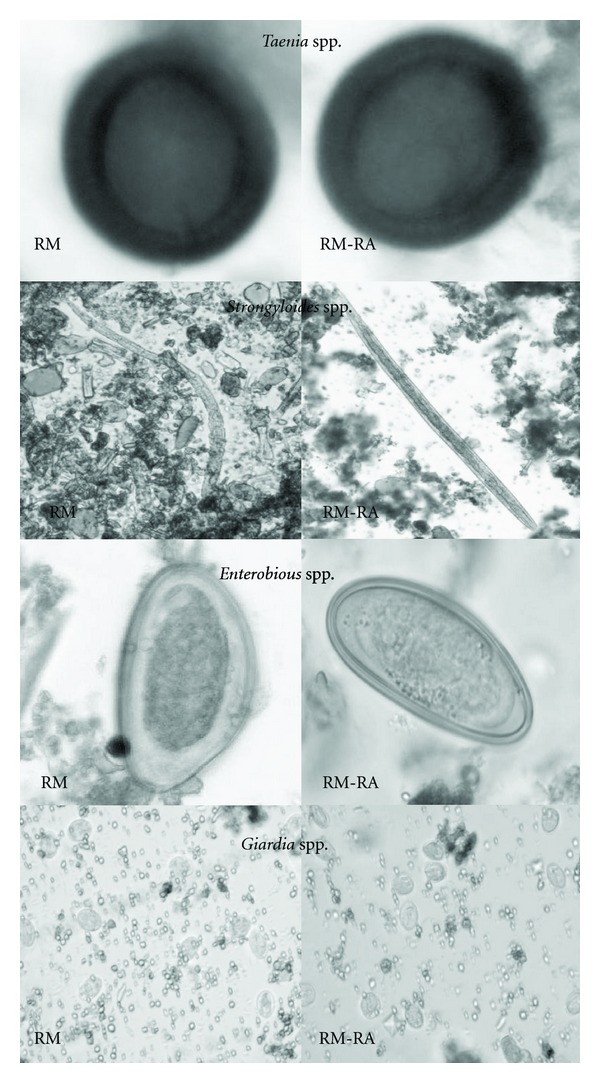
Qualitative Comparison of some eggs, cysts, and larvae found by Ritchie's methods (RMs) and Ritchie's methods modified by Régis Anécimo (RM-RA).

A qualitative comparison of the images obtained from the cysts, eggs and larvae identified by Ritchie's method and Ritchie's method modified by Régis Anécimo is presented in Figure 1. It is evidenced by observing the images that the modifications introduced in the traditional method permitted the satisfactory identification of the parasites when compared with the traditional method. Neutral detergent works as a surfactant dispersing agent, solubilizing the fat present in fecal matter, allowing the parasite structures identification, without morphological distortions.

Since the introduction of Ritchie's Method in 1948 [9], many laboratories have chosen the formalin-ether concentration procedure for parasitological exams in fecal specimens. However, taking into account the acknowledged dangers caused by exposure to ether and formaldehyde, research was initiated to develop techniques as efficient as Ritchie's Method [13, 14].

In 1979, it was developed a method that replaced diethyl ether by ethyl acetate with no morphological distortions or alterations, evidencing an equal or higher concentration of organisms than with diethyl ether [15]. In general, ethyl acetate seemed to increase the efficiency of the procedure both in terms of the number of organisms and the range of specimens recovered. Ethyl acetate was more effective than diethyl ether concentrating cysts of *Giardia* spp. and *H. nana*, which frequently are not recovered by the original method.

The effectiveness of ethyl acetate is similar to that of ether in the recovery of organisms, and it also presents the advantage of being less dangerous than ether. According to these studies, ethyl acetate seemed to be an efficient and less toxic solvent for routine use. In laboratories where the use of ether is prohibited or dangerous, ethyl acetate has been suggested as a substitute [15]. However, it is a harmful and irritating product to the skin and eyes and can cause burns. Prolonged and repetitive contact can aggravate these effects, and can damage skin, mucosa, and respiratory system [16].

Ethyl acetate also presented some other disadvantages: the thicker interface layers of ethyl acetate were difficult to remove and presented small bubbles, so that the reagent remained insoluble beneath the cover, obscuring small organisms. Therefore, it was verified that the method presents residues on the blades that difficult the identification of parasites and increase reading time.

Some authors investigated a d-Limonene (Hemo-De/4-isopropenil-1-metil-ciclohexano) solvent that presents a specific gravity and water solubility similar to ethyl acetate [17]. In that study, parasitological concentration methods were compared, using ether, ethyl acetate, or d-Limonene. The efficacy of the methods that used d-Limonene or ethyl acetate was equivalent in the concentration of intestinal parasites. However, the authors highlighted the use of d-Limonene because it is not toxic and also biodegradable. In addition, it represents an economy of 75% when compared to the method that combines formaldehyde and ethyl acetate [17].

More recently, a method that used formalin-polysorbate 20 (Tween 20) was published. It presented better results than the procedure with formalin ether. The method that uses formalin polysorbate is significantly superior for the identification of eggs of* Ascaris lumbricoides*; it also shows similar sensitivity for the identification of *Giardia lamblia*, *Trichuris trichiura*, *Entamoeba coli*, and *Entamoeba histolytica*. However, it was less effective in the identification of protozoa like *Endolimax nana*, and *Blastocystis hominis* [18]. Another disadvantage of this method was the need to use formalin, only replacing ether by polysorbate.

The modification of Ritchie's method is based on the strategy of eliminating chemical products. Many studies in literature modified the traditional method and demonstrated excellent efficiency for identification as well as for the reduction of chemical products. However, according to the bibliographic review developed during the planning and realization of the present study, no method has managed to fully eliminate the chemical products from the traditional method (Ritchie's method).

Ritchie's method modified by Régis Anécimo presents a great advantage in relation to the other methods because, besides eliminating the use of all chemical substances used in Ritchie's method, it also presented results comparable to those obtained through the traditional method, including the identification of *Strongyloides stercoralis*, which presents thermohydrotropism, typical of nematode larvae [19]. Therefore, Ritchie's method modified by Régis Anécimo can be used for parasitological diagnosis in clinical analysis laboratories.

## 4. Conclusion

The development of analytical diagnosis methods that minimize the use of chemical products like ether and formaldehyde represents an important tool to prevent occupational diseases among exposed professionals, as well as to preserve environmental quality by using the clean techniques. On the other hand, the development of techniques that minimize economic costs is very relevant, especially for developing countries, where the prevalence of parasitic diseases is higher and the financial resources destined for prophylaxis, control, and diagnosis are more limited.

## Figures and Tables

**Table 1:** Parasites percentage quantified by traditionally Ritchie's method and Ritchie's method modified by Régis Anécimo.

	Ritchie's method (%)	Ritchie's method modified by Régis Anécimo (%)
*Entamoeba coli*	36	28
*Giardia lamblia*	20	24
*Strongyloides stercoralis*	16	20
*Taenia *spp*. *	12	12
*Ascaris lumbricoides*	10	11
*Enterobius vermicularis*	4	4
*Schistosoma mansoni*	4	4
